# Understanding the drivers of sensitive behavior using Poisson regression from quantitative randomized response technique data

**DOI:** 10.1371/journal.pone.0204433

**Published:** 2018-09-28

**Authors:** Meng Cao, F. Jay Breidt, Jennifer N. Solomon, Abu Conteh, Michael C. Gavin

**Affiliations:** 1 Department of Statistics, Colorado State University, Fort Collins, Colorado, United States of America; 2 Department of Human Dimensions of Natural Resources, Colorado State University, Fort Collins, Colorado, United States of America; 3 School of Geography, Environment and Earth Sciences, Victoria University of Wellington, Wellington, New Zealand; Temple University, UNITED STATES

## Abstract

Understanding sensitive behaviors—those that are socially unacceptable or non-compliant with rules or regulations—is essential for creating effective interventions. Sensitive behaviors are challenging to study, because participants are unlikely to disclose sensitive behaviors for fear of retribution or due to social undesirability. Methods for studying sensitive behavior include randomized response techniques, which provide anonymity to interviewees who answer sensitive questions. A variation on this approach, the quantitative randomized response technique (QRRT), allows researchers to estimate the frequency or quantity of sensitive behaviors. However, to date no studies have used QRRT to identify potential drivers of non-compliant behavior because regression methodology has not been developed for the nonnegative count data produced by QRRT. We develop a Poisson regression methodology for QRRT data, based on maximum likelihood estimation computed via the expectation-maximization (EM) algorithm. The methodology can be implemented with relatively minor modification of existing software for generalized linear models. We derive the Fisher information matrix in this setting and use it to obtain the asymptotic variance-covariance matrix of the regression parameter estimates. Simulation results demonstrate the quality of the asymptotic approximations. The method is illustrated with a case study examining potential drivers of non-compliance with hunting regulations in Sierra Leone. The new methodology allows assessment of the importance of potential drivers of different quantities of non-compliant behavior, using a likelihood-based, information-theoretic approach. Free, open-source software is provided to support QRRT regression.

## Introduction

### Background and related work

Sensitive behaviors are those that are non-compliant with rules or regulations or are socially unacceptable. Sensitive behaviors are relevant to a variety of fields, including health sciences (e.g., abortion, illicit drug use, sexual activity), natural resource management (e.g., poaching of flora and fauna), business (e.g., tax evasion, insider trading), and education (e.g., cheating on exams). Although widespread, such behaviors are typically challenging to research, but understanding the behavior is paramount to creating effective interventions for the benefit of society at large. Successful interventions often require knowledge of who is engaged in the sensitive behavior, what the individuals are doing, where the sensitive behaviors take place, and why the individuals are engaged in the sensitive behaviors [1]. However, methodological constraints hamper collection of accurate data on such behaviors because participants are unlikely to disclose sensitive behaviors for fear of retribution or due to social undesirability.

Indirect survey methods allow researchers to gather information on sensitive behavior without the threat of implicating respondents [1]. Indirect methods for studying sensitive behavior include the randomized response technique (RRT; [2]), which provides anonymity to interviewees who answer sensitive questions. The original RRT has been modified by researchers (e.g., [3–5]) and applied in many contexts to help understand sensitive behaviors. See Fox and Tracy [6] or Chaudhuri and Mukerjee [7] for overviews of such methods and [8] for validation via a meta-analysis of randomized response studies. Use of RRT in surveys has been shown to increase a respondent’s proclivity to respond to questions about the sensitive behavior, as well as to increase the likelihood that a respondent provides accurate responses [8–11]. This method has shed light on sensitive behaviors in the fields of health sciences, natural resource management, business, education and political sciences [8]

The standard RRT approach uses a randomizing device, such as a coin or die, to determine the question a respondent answers. One or more questions are innocuous while another focuses on the sensitive behavior. The interviewer has no way of knowing which question the respondent is answering, thereby ensuring anonymity and increasing response rates and accuracy of responses provided. In this paper, we focus on nonnegative count data obtained via a modification of the technique referred to as the quantitative randomized response technique (QRRT) [5], which allows researchers to understand prevalence of a sensitive behavior in a community or society (e.g., [10]), as well as estimates of the frequency or quantity of the sensitive behavior (e.g., [4, 5]).

A major gap with the use of RRT has been in answering questions concerning drivers of non-compliance—the “why” question [1]. This is an essential question to investigators as it is typically critical when designing effective interventions to address non-compliance. Statistically, this corresponds to building and testing regression models for randomized response data. Logistic regression models for binary randomized response data are treated in [12] by recognizing the structure as a generalized linear model with a particular link function. Regression models are also developed in [12] for multi-category randomized response data, when the vector-valued observation comes from multiple randomized response questions. Another approach to inference with multiple sensitive questions is to sum the randomized responses; [13] and [14] develop regression models for such sum scores, including one based on zero-inflated Poisson regression. Some R packages [15, 16] have been developed to support this regression methodology.

However, to the best of our knowledge, regression methodology has not been developed for count data from QRRT [5]. We develop a methodology for Poisson regression with QRRT data, based on maximum likelihood implemented via the EM algorithm [17]. We implement the methodology in a freely-available R package, by adapting existing software for generalized linear models. Further, we provide an asymptotic theory to support estimation and testing of models. In particular, we derive the Fisher information matrix in this setting and use it to obtain the asymptotic variance-covariance matrix of the regression parameter estimates. Simulation results illustrate the quality of the asymptotic approximations. Using a case study of non-compliance with natural resource regulations [18], we demonstrate our new statistical approach to examine drivers of sensitive behavior.

### Case study: Non-compliance with hunting regulations in Sierra Leone

To demonstrate the utility of this new analytical approach, we examine the relative effects of different hypothesized drivers of non-compliant resource use activities inside the Western Area Peninsula Forest Reserve (WAPFR) in Sierra Leone. WAPFR comprises 175 km^2^ located between the Atlantic Ocean to the west and south, the capital city of Freetown 5km to the north, and a low-lying plain to the east. WAPFR is an important site for conservation in Sierra Leone because of the biodiversity it protects, including numerous endemic and highly threatened species, and also due to ecosystem services the reserve provides to 50 surrounding communities, including the main water source for Freetown’s 1.5 million residents. Communities neighboring WAPFR are home to all 17 of the country’s ethnic groups, which rely on gardening, small-scale businesses, sand extraction, fishing, and hunting for subsistence. Resource extraction is strictly prohibited inside WAPFR, but illegal hunting is a major threat [18–20].

The case study reported here was part of a larger examination of non-compliance in WAPFR (see [18–20]). We randomly selected 842 households (sampling every other household on a street) in eight communities that had similar numbers of households (100–500 households each). Coauthor Abu Conteh, a citizen of Sierra Leone, carried out the field research. Conteh surveyed heads of households in Krio (the lingua franca of Sierra Leone). Survey questionnaires can be found in Conteh, 2010 [21] (Appendices IV a & b) and are reproduced in S2 File for convenient reference. Ninety-eight percent of households answered all questions posed. We followed the standard research ethics procedures for the region at the time the research was conducted. This included obtaining permission from community leaders, and all respondents gave verbal consent (written consent was not used due to illiteracy rates in some of the communities sampled) to participate in the research. We did not record any information that could be used to identify individual respondents. Ethics approval to conduct the research was obtained from Victoria University of Wellington (Approval No. 15521).

We used the QRRT ([5, 22]) to estimate quantities of illegal hunting (see [18] for additional details). We recorded information on hunting activities over a nine-month period anchored by two widely known dates (New Years Day (January 1st) and Eid Ul Adha (October 1st) to reduce recall bias.

We designed and constructed a sealed, transparent, round bottomed container to serve as the randomizing device for QRRT. The container had a narrow neck that could only house one ball at a time. We placed 25 orange and 25 green balls into the container. Green balls had numbers from a known distribution painted on them [22]. Each respondent first turned their back on the interviewer and shook the container. If green fell into the neck of the container, the respondent read the number off the ball. If an orange ball fell into the neck, the respondent provided a numerical answer to the sensitive question the interviewer had posed prior to initiation of the exercise. The interviewer had no way of knowing whether the number stated by the respondent was innocuous (i.e. the number from a green ball) or was referring to the sensitive question (i.e. how many times per month on average did someone from the household hunt inside the reserve with the use of traps during the nine-month study period?). By ensuring anonymity in this way, QRRT encourages more truthful answers to questions regarding sensitive behavior [22]. However, as we outline below, because the researcher knows the probability of a respondent choosing a green or orange ball, as well as the distribution of numbers written on the green balls, estimates can be made of the quantities of sensitive behavior being conducted by different sectors of the populations.

Compliance with natural resource use regulations may be driven by a wide variety of potential factors [23–27]. To demonstrate the new analytical approach for the analysis of QRRT data, we compare the relative support for different hypothesized drivers of non-compliance with conservation regulations in Sierra Leone using an information theoretic approach. Specifically, we construct latent Poisson regression models that describe the effects on the amount of illegal trapping in WAPFR of perceived enforcement of the regulations, perceived resource rarity, access to alternative livelihoods, and other factors. We then fit and test these models using our new QRRT regression methodology.

## Methods

### Probability model

Let *T*_*i*_ denote the true count of the sensitive behavior, let *z*_*i*_ > 0 denote a known offset, and let ***x***_*i*_ = (*x*_*i*1_, …, *x_ip_*)′ denote a *p*-vector of known covariates for the *i*th individual, *i* = 1, …, *n*. Assume that

$$\begin{array}{ccc} T_{i} & ∼ & \text{independent}\;\text{Poisson}(z_{i}\lambda_{i})\\ \ln(\lambda_{i}) & = & x_{i}^{\prime }\beta \end{array}$$

where

$$\begin{array}{c} \text{P}[T_{i}=t|\lambda_{i}]=\frac{e^{-z_{i}\lambda_{i}}{\left(z_{i}\lambda_{i}\right)}^{t}}{t!}=\pi_{i}\left(t|\beta \right) \end{array}$$ (1)

for *t* = 0, 1, 2, …, and ***β*** is a *p*-vector of unknown parameters.

The ${\left\{T_{i}\right\}}_{i=1}^{n}$ are not observed directly, but are masked through QRRT [5] as described in the Sierra Leone example. Let *m* be a known positive integer and let *b*(*r*) denote a completely known probability mass function on the integers 0, 1, …, *m*, *m* + 1. Let *N* denote the total number of balls and assume that *Nb*(0), *Nb*(1), …, *Nb*(*m* + 1) are all integers. Then *Nb*(0) balls are marked 0, *Nb*(1) balls are marked 1, …, *Nb*(*m*) balls are marked *m*, and *Nb*(*m* + 1) balls are blank. The *i*th interviewee selects a random integer *B*_*i*_ ∼ *b*(*r*) by selecting one of the balls. If *B*_*i*_ ≤ *m*, the ball is numbered and the interviewee’s response is the ball number, *R*_*i*_ = *B*_*i*_. If *B*_*i*_ = *m* + 1, the ball is blank and the interviewee’s response is the true count, *R*_*i*_ = *T*_*i*_. Since no one but the interviewee knows the value of *B*_*i*_, only the interviewee knows whether the response is a true value *T*_*i*_ or a randomized response *B*_*i*_, assuming *T*_*i*_ ≤ *m*. This requires some care in the choice of *m*, to ensure it is sufficiently large: any reported values larger than *m* are known to be true counts. The higher the ratio of blank balls to marked balls, $b\left(m+1\right)/\sum_{r=0}^{m}b\left(r\right)$, the higher the expected number of true responses and the more powerful the inference, but the lower the guarantee of anonymity. The lower the ratio of blank to marked, the lower the expected number of true responses, but the higher the guarantee of anonymity; see [5]. While the choice of the distribution *b*(*r*) is up to the researcher, it would be very difficult to optimize this choice without detailed information about the unknown distribution of true responses.

### Poisson regression via EM algorithm

If the ${\left\{T_{i}\right\}}_{i=1}^{n}$ were observed directly, inference could proceed via Poisson regression fitted by maximum likelihood. Since only the realized values ${\left\{r_{i}\right\}}_{i=1}^{n}$ of the random variables ${\left\{R_{i}\right\}}_{i=1}^{n}$ are observed, we use the Expectation-Maximization (EM) algorithm [17] to maximize the likelihood, by first augmenting with the unobserved values ${\left\{B_{i}\right\}}_{i=1}^{n}$.

If the ${\left\{B_{i}\right\}}_{i=1}^{n}$ values were known, we would discard all but the true data values, for which $1_{\left\{B_{i}=m+1\right\}}=1$, resulting in the complete-data log-likelihood

$$\begin{array}{c} \sum_{i=1}^{n}1_{\left\{B_{i}=m+1\right\}}\{-\ln\left(r_{i}!\right)-z_{i}\lambda_{i}+r_{i}\ln z_{i}+r_{i}\ln\lambda_{i}\}. \end{array}$$ (2)

The incomplete-data log-likelihood is the conditional expectation of (2) given the observed data and the current estimate of ***β***, denoted ***β***^(*k*)^:

$$\begin{array}{c} \sum_{i=1}^{n}\text{P}[B_{i}=m+1|\left\{R_{i}\right\}=\left\{r_{i}\right\},\beta^{\left(k\right)}]\{-\ln\left(r_{i}!\right)-z_{i}\lambda_{i}+r_{i}\ln z_{i}+r_{i}\ln\lambda_{i}\}\\ =\sum_{i=1}^{n}\omega_{i}^{\left(k\right)}\{-\ln\left(r_{i}!\right)-z_{i}e^{x_{i}^{\prime }\beta }+r_{i}\ln z_{i}+r_{i}x_{i}^{\prime }\beta \}, \end{array}$$ (3)

where the conditional probabilities ${\left\{\omega_{i}^{\left(k\right)}\right\}}_{i=1}^{n}$ are computed via Bayes’ rule as

$$\begin{array}{c} \omega_{i}^{\left(k\right)}=\frac{\pi_{i}(r_{i}|\beta^{\left(k\right)})b\left(m+1\right)}{b\left(r_{i}\right)1_{\left\{r_{i}<m+1\right\}}+\pi_{i}(r_{i}|\beta^{\left(k\right)})b\left(m+1\right)}. \end{array}$$ (4)

The EM algorithm then reduces to iterating the following steps across *k* to maximize the likelihood and obtain the maximum likelihood estimator (MLE) $\hat{\beta }$:

- **E-step**: compute weights from (4) under the current maximized model with parameters ***β***^(*k*)^.
- **M-step**: maximize the weighted log-likelihood (3) for Poisson regression.

### Asymptotic distribution and variance estimation

In derivations not described here, we have verified the regularity conditions in chapter 2 of Fahrmeir and Tutz [28], establishing that the MLE is asymptotically normally distributed as *n* → ∞. Thus, in large samples,

$$\begin{array}{c} \hat{\beta }\;\text{is}\;\text{approximately}\;N(\beta ,I^{-1}(\beta )), \end{array}$$

where ***β*** is the vector of true regression coefficients and $I^{-1}(\beta )$ is the inverse of the Fisher information matrix. We derive the Fisher information matrix in the supplemental material, S1 Appendix. The asymptotic covariance matrix $I^{-1}(\beta )$ is then estimated by plugging in the MLE, $\hat{\text{Var}}(\hat{\beta })=I^{-1}(\hat{\beta })$.

### Hypothesis testing and model selection

The log-likelihood $\ell (\beta ;{\left\{r_{i}\right\}}_{i=1}^{n})$ derived in the supplemental material, S1 Appendix:, can be used in hypothesis testing and model selection. First, let ***β***_full_ be a vector of *p* parameters for a full model that fits the data well. Let ***β***_reduced_ be a vector of *q* parameters for a nested (reduced) model within the full model (that is, a model obtained by setting *p* − *q* of the parameters in ***β***_full_ equal to zero). To test the null hypothesis that the reduced model fits the data equally as well as the full model, we compute the likelihood ratio test statistic

$$\begin{array}{c} W=-2\ell ({\hat{\beta }}_{\text{reduced}};{\left\{r_{i}\right\}}_{i=1}^{n})+2\ell ({\hat{\beta }}_{\text{full}};{\left\{r_{i}\right\}}_{i=1}^{n}), \end{array}$$ (5)

where ${\hat{\beta }}_{\text{full}}$ and ${\hat{\beta }}_{\text{reduced}}$ are the MLE’s for the full and reduced models, respectively. Standard asymptotic theory shows that for *n* large, *W* has an approximate $\chi_{p-q}^{2}$ distribution, the chi-squared distribution with *p* − *q* degrees of freedom. We reject the reduced model in favor of the full model if the test statistic is large (e.g., [29]).

The maximized log-likelihood can also be used to compare models that need not be nested, via Akaike’s information criterion (AIC, [30]). For a model with *p* parameters ***β***,

$$\begin{array}{c} \text{AIC}=-2\ell (\hat{\beta };{\left\{r_{i}\right\}}_{i=1}^{n})+2p. \end{array}$$

We use AIC to rank models for comparison, with small AIC being the best. Models are competitive with one another if their AIC values differ by less than two.

### Numerical implementation

Maximization of the weighted log-likelihood (3) for Poisson regression can be accomplished with standard software, such as the R function glm, using case weights (4) obtained in the E-step. We developed custom code for fitting of these models, and have made it available as an R package called QRRT, freely downloadable from GitHub; see the supplemental material S1 Code for details.

We use multiple starting values and iterate each to convergence, assessed by checking the value of the score vector derived in S1 Appendix. We then choose the set of converged parameter estimates that yield the highest log-likelihood value. Standard errors (SE’s) for each estimated parameter ${\hat{\beta }}_{j}$ are calculated from diagonal elements of the estimated Fisher information matrix (S1 Appendix). The *t*-statistic is calculated as $t_{j}={\hat{\beta }}_{j}/{\text{SE}}_{j}$ and the corresponding *p*-value is the probability that the absolute value of a standard normal random variable is greater than or equal to |*t*_*j*_|; that is, the probability under the asymptotic distribution of obtaining a statistic this extreme or more extreme under the null hypothesis that the true *β*_*j*_ coefficient is zero. The code returns AIC and the maximized log-likelihood, so that non-nested models can be compared, and nested models can be tested.

## Results

### Monte Carlo results

We illustrate the methodology and the quality of the asymptotic approximations via a Monte Carlo experiment using our R package QRRT. Details on reproducing results of this simulation experiment are given in the supplemental material, S1 Code.

We consider a setting in which *n* = 400 true counts are generated independently as *T*_*i*_ ∼ Poisson(λ_*i*_) with

$$\begin{array}{c} \ln\lambda_{i}=\beta_{0}+\beta_{1}x_{i1}+\beta_{2}x_{i2}+\beta_{3}1_{\left\{x_{i3}=B\right\}}+\beta_{4}1_{\left\{x_{i3}=C\right\}}+\beta_{5}x_{i1}x_{i2}, \end{array}$$ (6)

where *x*_1*i*_ and *x*_2*i*_ are continuous predictors and *x*_3*i*_ is a categorical predictor with levels “A”, “B”, and “C”, and “A” is the baseline level. We set

$$\begin{array}{c} \left(\beta_{0},\ldots ,\beta_{5}\right)=\left(1.5,1.0,-0.5,0.4,0.3,0.2\right). \end{array}$$

Next, we simulate ${\left\{B_{i}\right\}}_{i=1}^{n}$ as independent and identically distributed from the same *b*(*r*) distribution as in Conteh [18], with *m* = 8 and

$$\begin{array}{c} (b(0),b(1),\ldots ,b(8),b(9))=\frac{1}{50}\left(6,7,4,2,2,1,1,1,1,25\right). \end{array}$$ (7)

Observations are then *R*_*i*_ = *B*_*i*_ if *B*_*i*_ ≤ *m* = 8 and *R*_*i*_ = *T*_*i*_ if *B*_*i*_ = *m* + 1 = 9.

For our Monte Carlo experiment, we fixed ${\left\{\left(x_{1i},x_{2i},x_{3i}\right)\right\}}_{i=1}^{400}$ and, over 1000 independent realizations, simulated {*T*_*i*_} using the model (6) and {*B*_*i*_} and {*R*_*i*_} as described. We fitted each of the 1000 simulated data sets both with the true model (6), and with the larger-than-necessary model with all two-way interactions,

$$\begin{array}{c} \begin{array}{cc} & \beta_{0}+\beta_{1}x_{i1}+\beta_{2}x_{i2}+\beta_{3}1_{\left\{x_{i3}=B\right\}}+\beta_{4}1_{\left\{x_{i3}=C\right\}}+\beta_{5}x_{i1}x_{i2}\\ & +\beta_{6}x_{1i}1_{\left\{x_{i3}=B\right\}}+\beta_{7}x_{1i}1_{\left\{x_{i3}=C\right\}}+\beta_{8}x_{2i}1_{\left\{x_{i3}=B\right\}}+\beta_{9}x_{2i}1_{\left\{x_{i3}=C\right\}}, \end{array} \end{array}$$ (8)

in which *β*_6_ = ⋯ = *β*_9_ = 0.

For each simulated realization and both fits, we recorded the vector of estimated coefficients and the inverse Fisher information evaluated at the estimated parameters. We then compared the average regression coefficient vector over the 1000 Monte Carlo replicates to the vector of true coefficients, to assess the quality of the point estimation, and the empirical covariance matrix over the 1000 Monte Carlo replicates to the asymptotic covariance matrix given by the inverse Fisher information at the true values, to assess the quality of the variance approximation. Further, we compared the average estimated inverse Fisher information to the empirical covariance matrix, to assess the quality of the variance estimators.

Results are given in Table 1 and show that the asymptotic approximations are excellent. For both the true additive model and the larger interaction model, the MLE’s are approximately unbiased, their variances are well-approximated by diagonal elements of inverse Fisher information, and the estimated variances obtained by plugging MLE’s into inverse Fisher information are nearly unbiased for the true variances.

**Table 1:** Simulation results. True coefficients, estimated parameters, Monte Carlo standard error, inverse Fisher information matrix evaluated at estimated parameters and inverse Fisher information matrix at the true value. All parameters are calculated based on 1000 Monte Carlo replicates with sample size equals to 400.

	True *β*	True Model				Interaction Model			
		$\hat{\beta }$	Monte Carlo S.E.	Average estimated S.E.	Inverse Fisher at true value	$\hat{\beta }$	Monte Carlo S.E.	Average estimated S.E.	Inverse Fisher at true value
*β*_0_	1.5	1.4951	0.0815	0.0791	0.0789	1.4899	0.1489	0.1464	0.1456
*β*_1_	1.0	1.0037	0.0690	0.0676	0.0675	1.0067	0.1446	0.1405	0.1400
*β*_2_	-0.5	-0.5005	0.0651	0.0646	0.0645	-0.5008	0.0750	0.0733	0.0728
*β*_3_	0.4	0.3987	0.0514	0.0508	0.0507	0.3998	0.1874	0.1818	0.1808
*β*_4_	0.3	0.3007	0.0523	0.0501	0.0500	0.3031	0.1846	0.1818	0.1808
*β*_5_	0.2	0.2003	0.0626	0.0623	0.0622	0.2006	0.0648	0.0631	0.0632
*β*_6_						0.0000	0.1800	0.1720	0.1715
*β*_7_						-0.0014	0.1776	0.1734	0.1726
*β*_8_						-0.0002	0.0523	0.0512	0.0510
*β*_9_						-0.0008	0.0539	0.0517	0.0520

Finally, for each simulated realization we tested the null hypothesis that the true model (6) suffices,

$$\begin{array}{c} H_{0}:\;\beta_{6}=\cdots =\beta_{9}=0, \end{array}$$

against the alternative that the larger, two-way model (8) is necessary. These hypotheses were compared via a likelihood ratio test, computed as

$$\begin{array}{c} -2\ln(\text{likelihood}\;\text{of}\;\text{true}\;\text{model})+2\ln(\text{likelihood}\;\text{of}\;\text{full}\;\text{two-way}\;\text{model}) \end{array}$$ (9)

and compared to the *χ*^2^ distribution with 4 degrees of freedom, rejecting *H*_0_ for large values of the test statistic. Since the null hypothesis is true in each simulated realization, the *p*-values should theoretically follow a uniform distribution. The empirical results (not shown here) are consistent with the uniform distribution. In particular, the empirical proportion of rejections is 0.047 at the 0.05 significance level and 0.092 at the 0.10 significance level.

### Application to poaching in Sierra Leone

We applied our method to responses to the question “how many times per month on average did someone from the household hunt inside the reserve with the use of traps during the nine-month study period?” Instrumental models of compliance [23, 25, 31] posit that compliance is primarily driven by factors external to the individual, including the probability of being caught and convicted. To test for the effects of perceived enforcement we asked respondents if they knew that a protected area existed neighboring their community, if reserve personnel restricted the activities allowed inside the protected area, if reserve personnel patrolled the reserve, if the personnel were efficient in their enforcement duties, if conservation personnel were quick to apprehend those engaged in non-compliant activities in the reserve, and if those caught were punished.

Non-compliance may also be influenced by other perceived costs and benefits of a particular behavior. For example, if resources are rare, the efforts needed to obtain them may outweigh any benefits received. To test the effect of perceived rarity, we asked respondents about the rarity of targeted species. Similarly, we tested for the effect of household size (the number of people living in the household), as larger households may require more resources, which would increase the likelihood of violating hunting regulations while searching for food.

In addition, alternative livelihoods may reduce the need for subsistence-based hunting practices [32]. We stratified our sample based on access to alternative livelihoods.

Urban centers can both drive more illegal hunting by providing markets for bushmeat, or wage labor in urban areas may reduce illegal hunting by offering alternative livelihoods [32–35]. Therefore, we surveyed communities with both high and low access to the main urban center of Freetown. Similarly, we might predict less illegal hunting in locations with better ocean access, due to the presence of alternative marine-based livelihoods [36]; and therefore we surveyed communities with both direct and no access to the ocean. Sierra Leone’s civil war (1992–2002) displaced millions of people. Many of the displaced settled in communities near Freetown. Communities surrounding WAPFR vary widely in terms of the proportion of residents that arrived as internally displaced people during Sierra Leone’s civil war. Many of the internally displaced do not have access to suitable land for agriculture or other alternative livelihoods to meet basic needs, which can lead to increases in resource extraction rates from the reserve. We surveyed communities with either no internally displaced people or substantial populations of internally displaced people. We then included community dummy variables in our models to examine the effect of context, including access to alternative livelihoods.

The normative view argues that compliance is more internally driven by perceived behavioral norms [23, 36–38]. Here we explore the effects of descriptive norms, which involve a person’s perceptions of the prevalence of a behavior [39]. Based on descriptive norms, we would hypothesize that people will be more likely to violate regulations if they believe many of their peers are also non-compliant. To test for these normative effects we asked respondents if people from their community hunted inside the reserve, and if they thought people from outside the community hunted in the reserve.

Finally, hunting requires specialized knowledge of the local ecosystem and of target species. Ecological knowledge can accumulate over time as hunters compile more first-hand experience, and several ethnobiological studies have found residence time to be positively correlated with increased natural resource use [40, 41]. To the contrary, formal education has often been significantly linked to lower levels of ecological knowledge and subsistence resource use [42–45]. Based on these prior findings, we tested for the effects of both formal education level and local residence time in our models.

Summarizing, the set of hypothesized drivers and corresponding covariates is given in Table 2. Among these covariates, all of the Yes-No-Don’t Know variables were converted to Yes indicators, and all agreement scales (1 = Strongly Agree, …, 5 = Strongly Disagree) were converted to Agreement (Agree or Strongly Agree) indicators. The data set was then restricted to records with non-missing values for all of the above variables, to ensure comparability across fitted models. There are *n* = 662 complete records in this data set. These data are available in the supplemental material, S1 Dataset, with variables described in the data dictionary, S1 File.

**Table 2:** Drivers and covariates. Hypothesized drivers of non-compliant behavior and corresponding measured covariates in the Sierra Leone dataset.

Driver	Covariates
**perceived enforcement**	knowledge of protected area, no perceived restriction on extraction, perceived efficiency of conservation personnel, perceived patrols, perceived rapid detention, perceived punishment
**perceived rarity**	
**household size**	
**alternative livelihoods**	rural, seaside, displaced
**descriptive norms**	residents hunted, outsiders hunted
**residence time**	
**formal education**	

We fitted a series of models corresponding to the hypothesized drivers in Table 2. Each of the models included an intercept. All covariates for a hypothesized driver were either simultaneously included or excluded from a model; for example, all six covariates corresponding to perceived enforcement were either in or out of a given model. Hence, with seven drivers there were 2^7^ = 128 possible additive models for consideration, with the largest model including the intercept and all seven drivers, and the smallest (null) model including only the intercept.

We used our code to fit all of these models, plus three sets of additional models, each with 128 subset models: (1) all subsets of the seven drivers, with alternative livelihoods replaced by (alternative livelihoods)^2^, meaning the three community variables plus all three of their two-way interactions; (2) all subsets of the seven drivers, but with the six variables of perceived enforcement replaced by the single variable “Efficient Conservation: perceived efficiency of conservation personnel”; (3) all subsets of the seven drivers, but with both alternative livelihoods replaced by (alternative livelihoods)^2^ and perceived enforcement replaced by Efficient Conservation. We computed AIC for all of these subset models and determined minimum AIC within each model class (see Table 3). Based on these computations, we restricted attention to the model class Efficient Conservation + (alternative livelihoods)^2^.

**Table 3:** Minimum AIC for four different model classes. Minimum AIC over all 128 subset models in each model class. All models are fitted to randomized responses based on the EM algorithm with 20 different random starting values to avoid convergence to local modes.

Model Class	Minimum AIC
Efficient Conservation + alternative livelihoods	2728.699
Efficient Conservation + (alternative livelihoods)^2^	2680.613
perceived enforcement + alternative livelihoods	2729.034
perceived enforcement + (alternative livelihoods)^2^	2688.773

Within this model class, we computed ΔAIC as AIC minus minimum AIC, and focused on the nine optimal models with ΔAIC < 2 (see Table 4). We found support for some hypotheses we tested and not for others in this set. In addition, none of the individual hypothesized factors alone explains the variation in frequencies of illegal hunting. The ΔAIC value of models containing just individual factors are between 31.231 and 118.455. Instead, all optimal models contained a combination of different factors.

**Table 4:** Top models with ΔAIC less than 2 for Efficient Conservation + (alternative livelihoods)^2^. ΔAIC, maximum likelihood estimates for models fitted to randomized responses. All model fits are based on the EM algorithm with 20 different random starting values to avoid convergence to local modes.

	1	2	3	4	5	6	7	8	9
(Intercept)	-0.469	-0.338	-0.460	-0.338	-0.589	-0.319	-0.342	-0.454	-0.451
EfficientConservation	-3.261	-3.316	-3.127	-2.716	-3.213	-2.879	-3.200	-3.248	-3.264
AnimalsRare		-0.218					-0.201		-0.212
HouseholdSize								-0.002	
HighDisplace	0.691	0.671	0.670	0.668	0.713	0.685	0.656	0.688	0.690
Rural	1.404	1.489	1.500	1.664	1.414	1.559	1.559	1.395	1.505
Seaside	1.814	1.762	1.842	1.915	1.790	1.900	1.802	1.810	1.743
Rural:HighDisplace	-0.288	-0.308	-0.354	-0.493	-0.315	-0.397	-0.355	-0.275	-0.340
Seaside:HighDisplace	-0.887	-0.781	-0.886	-0.909	-0.854	-0.909	-0.800	-0.877	-0.751
Seaside:Rural	-2.361	-2.408	-2.382	-2.434	-2.329	-2.457	-2.424	-2.357	-2.381
OutsidersHunted			-0.283	-0.366			-0.273		
ResidentsHunted			0.282	0.342			0.280		
Residencetime	0.007	0.008	0.006		0.007		0.007	0.007	0.008
Education.level					0.038				0.036
AIC	2680.613	2681.220	2681.764	2681.868	2681.916	2682.067	2682.500	2682.591	2682.601
ΔAIC	0	0.607	1.151	1.255	1.303	1.454	1.887	1.978	1.988

Other likelihood-based criteria could be applied, such as the Bayesian Information Criterion (BIC) [46]. AIC and BIC both allow for model selection in large model spaces, but using different approaches: AIC efficiently selects a good approximating model in the model space, while BIC consistently estimates the true model if a true model is in fact in the model space. We computed BIC for all 128 models in the same model class as considered for Table 4. As expected, BIC tends to prefer smaller models, but model 6 and model 1 in Table 4 are the first and second model selected based on BIC.

All optimal models included a large, negative coefficient for perceived enforcement, indicating that higher levels of enforcement may serve as a critical deterrent against illegal hunting in WAPFR (Table 4), as has been found in a wide variety of other protected areas. This outcome has clear policy and management implications; however, the potential to increase enforcement may be limited in Sierra Leone. The country faces many fiscal challenges, and conservation capacity in WAPFR has yet to recover to levels seen prior to the civil war [20].

Three of the nine optimal models also point to the importance of normative influences on the amount of non-compliance (Table 4). We found that community members were more likely to engage in illegal hunting when they believed their neighbors in the same community were also doing so (positive coefficients in Table 4). Norms have been shown to influence compliance with conservation regulations and to shape natural resource use patterns across a broad range of contexts from recreational fishing in New Zealand [23] to rangeland management in Mongolia [47]. Management interventions can influence norms, but care must be taken as the introduction of new rules and regulations can undermine long-standing norms and drive greater non-compliance [48]. One promising approach is community-based social marketing, which can use social norms as the center piece of persuasive behavior-change communication campaigns [49].

We also found that, contrary to initial hypotheses, respondents were less likely to hunt illegally if they perceived outsiders were hunting in the reserve (negative coefficients in Table 4). One possible reason for this apparent contradiction is that the effects of descriptive norms are moderated by group identity. Specifically, when an individual perceives a group to be more similar to themselves, the individual may identify more closely with the group, and this may increase the influence of descriptive norms on the individual’s behavior [50]. In other words, individuals should be more likely to participate in a behavior that is common among a group they identify with (in this case their home community) than a behavior common in a less similar group (in this case outsiders). This could explain why the perceived behaviors of outsiders would have less effect on the amount of non-compliance than the behaviors of community members. However, we found that the effect of outsiders was as strong as that of community members, but in the opposite direction: perceptions of hunting by outsiders correlates with less hunting by respondents and perceptions of hunting by community members correlates with more hunting. The effect of outsider’s behaviors may instead be explained by the history of the region. During the war, combatants frequented the forest inside the reserve, and local people may still harbor memories that associate the forest with zones of active combat [19]. Therefore, increased activity of outsiders in the reserve may provide local people with ample reason to avoid the area.

All nine optimal models also included community variables (Table 4). As described above, we had included community as a variable in our models as a proxy for access to alternative livelihoods. Some of the results support the idea that increased availability of alternative livelihoods can reduce resource use and non-compliance with conservation regulations. For example, rural communities, with less access to labor markets in urban centers, tended to hunt more in the reserve (positive coefficients in Table 4). In addition, communities with a greater proportion of displaced people also hunted more. However, contrary to our hypotheses, we found more hunting to occur in seaside communities, despite their access to additional marine resources. Also, examining interaction effects among community types, further confounds the relationship between access to alternative livelihoods and frequency of hunting. For example, we would expect rural communities with many displaced people to have high rates of hunting, however, all of our optimal models found that these communities had lower rates of hunting (negative coefficient in Table 4 for interaction between rural and displaced). Overall, we see a significant difference in hunting rates among communities, but these differences cannot be explained by access to alternative livelihoods. Instead, other aspects of the local context not measured here must be driving these differences in hunting rates among communities.

Seven of the nine optimal models also contained residence time. The small coefficients (Table 4) indicate the smaller effect the variable had on the outcome. In all cases the longer a household had lived in a community, the greater the likelihood they had participated in illegal hunting. This corroborates prior findings of increases in the use of forest resources with longer residence times, which may be linked to the accumulation of ecological knowledge over time [40]. Three of the optimal models included perceived rarity, and supported the prediction that residents were less likely to participate in illegal hunting when they perceived animals to be rare in the reserve. Only two models included education, but contrary to prior studies [42–45] our results indicate that increases in formal education are associated with greater amounts of illegal hunting. However, some studies in Africa have found similar results using indirect questioning methods [51, 52]. In addition to the value of using an indirect questioning method such as QRRT, our finding might be explained by the links between hunting and bushmeat markets in the nearby capital of Freetown. Higher levels of education may assist some families in integrating with these markets, but further research is needed to confirm this possible link between education and hunting. Finally, only one model in the optimal set contained household size. The coefficient for the variable was small and surprisingly indicated that larger households would be slightly less likely to hunt illegally in the reserve. Although this finding is in contrast to theory, similar results have been recorded in Gabon in the case of hunting for bushmeat [53].

Although our models allow us to compare the relative importance of possible drivers of illegal hunting, the models still only explained a relatively small proportion of the variance in hunting rates. This is not surprising given that our aim was to use this case to demonstrate a new methodological approach and we did not attempt to measure all possible determinants of non-compliance. For this case, future research might include variables or models not tested here, but for which strong theoretical foundations exist. Possibilities for additional theories to test that have been found to be good predictions of conservation-related behavior in past studies include the theory of planned behavior, which posits that attitudes and perceived behavioral control, along with social norms all influence behavioral intentions [54], Bamberg and Moser’s [55] framework of pro-environmental behavior, and models of legitimacy, which include both measures of participation in decision-making as well as perceptions of the fairness of rules and enforcement outcomes [56, 57].

## Conclusion

The methods we present here provide a methodological blueprint for examining possible drivers of sensitive behaviors. Researchers across multiple disciplines are interested in understanding sensitive behaviors, and policy makers and program managers seek more effective means to reduce the frequency of a wide variety of sensitive behaviors. QRRT provides a means for gathering data on the frequency of sensitive behaviors while protecting respondent anonymity. The new analytical approach and tools we present here will allow researchers to explore drivers of a wide variety of sensitive behaviors using QRRT data.

## Supporting information

S1 Appendix Log-likelihood, score vector and Fisher information matrix. (PDF)

S1 Code R package for Poisson regression from QRRT data. (PDF)

S1 Dataset QRRT dataset for poaching in Sierra Leone. (CSV)

S1 File Data dictionary for the QRRT dataset, S1 Dataset. (CSV)

S2 File Survey questionnaires. (PDF)
