# Supplementary material for: Understanding the drivers of sensitive behavior using Poisson regression from quantitative randomized response technique data
Source: PLoS One. 2018 Sep 28;13(9):e0204433. doi: 10.1371/journal.pone.0204433 (PMC6161884; doi:10.1371/journal.pone.0204433)
Supplement: S1 Appendix — (PDF) [file pone.0204433.s001.pdf]

S1 APPENDIX: LOG-LIKELIHOOD, SCORE VECTOR AND FISHER INFORMATION MATRIX

MENG CAO, F. JAY BREIDT, JENNIFER N. SOLOMON, ABU CONTEH, MICHAEL C. GAVIN

The log-likelihood of the observations $\{r_i\}_{i=1}^n$ is given by

$$
\begin{aligned}
\ell\left(\boldsymbol{\beta}; \{r_i\}_{i=1}^n\right) &= \sum_{i=1}^n \ln \mathrm{P}\left[R_i = r_i\right] \\
&= \sum_{i=1}^n \ln\left\{\mathrm{P}\left[R_i = r_i \mid B_i = r_i\right]\mathrm{P}\left[B_i = r_i\right] + \mathrm{P}\left[R_i = r_i \mid B_i = m+1\right] b(m+1)\right\} \\
&= \sum_{i=1}^n \ln\left\{b(r_i) + \pi_i(r_i \mid \boldsymbol{\beta}) b(m+1)\right\} \qquad (1)
\end{aligned}
$$

where $\pi_i(r_i \mid \boldsymbol{\beta})$ is the Poisson probability mass function with mean $\mu_i = z_i \exp(\boldsymbol{x_i'\beta})$. Let $g(r_i) = b(r_i) + \pi_i(r_i \mid \boldsymbol{\beta}) b(m+1)$. Then the jth component of the score vector is

$$
\begin{aligned}
\frac{\partial \ell}{\partial \beta_j} &= \sum_{i=1}^n \frac{\partial}{\partial \beta_j} \ln\{g(r_i)\} = \sum_{i=1}^n \frac{1}{g(r_i)} x_{ij} b(m+1)\left(\frac{-e^{-\mu_i}\mu_i^{r_i+1}}{r_i!} + \frac{r_i \mu_i^{r_i} e^{-\mu_i}}{r_i!}\right) \\
&= \sum_{i=1}^n f_j(r_i)
\end{aligned}
$$

for $j = 1, 2, \ldots, p$.

The Fisher information matrix is defined as $\mathcal{I}(\boldsymbol{\beta}) = -\mathrm{E}\left(\partial^2 \ell / \partial \beta_j \partial \beta_k\right)$ for $j, k = 1, 2, \ldots, p$. Now

$$
\begin{aligned}
\frac{\partial^2 \ell}{\partial \beta_j \partial \beta_k} &= \sum_{i=1}^n \frac{\partial}{\partial \beta_k} f_j(r_i) \\
&= \sum_{i=1}^n \left[\{-f_j(r_i) f_k(r_i)\} + \frac{x_{ij} x_{ik} b(m+1)}{g(r_i) r_i!} \times \right. \\
&\left. \left\{e^{-\mu_i}\mu_i^{r_i+2} - (r_i+1)e^{-\mu_i}\mu_i^{r_i+1} + r_i^2 \mu_i^{r_i} e^{-\mu_i} - e^{-\mu_i} r_i \mu_i^{r_i+1}\right\}\right],
\end{aligned}
$$

so,

$$
\mathrm{E}\left(\frac{\partial^2 \ell}{\partial \beta_j \partial \beta_k}\right) = \sum_{i=1}^n \left[x_{ik} x_{ij}\left\{-b(m+1)\mu_i + b(m+1)\sum_{r=0}^m \frac{\pi(r) b(r)}{g(r)}(r - \mu_i)^2\right\}\right].
$$

Two special cases of the information matrix are of interest. If $b(m+1) = 0$, then no responses are true, and the data contain no information about the model parameters: $\mathrm{E}\left(\partial^2 \ell / \partial \beta_j \partial \beta_k\right) = 0$ for all j and k. If $b(0) = b(1) = \cdots = b(m) = 0$, then all responses are true, and the information matrix is that of ordinary Poisson regression.
