# Supplementary material for: Understanding the drivers of sensitive behavior using Poisson regression from quantitative randomized response technique data
Source: PLoS One. 2018 Sep 28;13(9):e0204433. doi: 10.1371/journal.pone.0204433 (PMC6161884; doi:10.1371/journal.pone.0204433)
Supplement: S1 Code — (PDF) [file pone.0204433.s002.pdf]

S1 CODE: R PACKAGE FOR POISSON REGRESSION FROM QRRT DATA

MENG CAO, F. JAY BREIDT, JENNIFER N. SOLOMON, ABU CONTEH, MICHAEL C. GAVIN

The simulation and empirical results of this paper were obtained using our `R` package `QRRT`. The `R` language and environment for statistical computing [1] is freely available and runs on many computing platforms (UNIX, Windows, MacOS). From within `R`, the `QRRT` package is downloadable via GitHub (install the package `devtools` first, if necessary) using the following commands:

```
library(devtools)
install_github("meca7653/QRRT")
library(QRRT)
```

The following reproducible example is included with the code and accessed with `help(QRRT)`. The example uses one simulated realization from the true, additive model

$$\ln \lambda_i = \beta_0 + \beta_1 x_{i1} + \beta_2 x_{i2} + \beta_3 \mathbf{1}_{\{x_{i3}=\mathrm{B}\}} + \beta_4 \mathbf{1}_{\{x_{i3}=\mathrm{C}\}} + \beta_5 x_{i1} x_{i2}, \tag{1}$$

as described in the simulation section. It fits those simulated data using the larger-than-necessary interaction model

$$\begin{aligned} &\beta_0 + \beta_1 x_{i1} + \beta_2 x_{i2} + \beta_3 \mathbf{1}_{\{x_{i3}=\mathrm{B}\}} + \beta_4 \mathbf{1}_{\{x_{i3}=\mathrm{C}\}} + \beta_5 x_{i1} x_{i2} \\ &+ \beta_6 x_{1i} \mathbf{1}_{\{x_{i3}=\mathrm{B}\}} + \beta_7 x_{1i} \mathbf{1}_{\{x_{i3}=\mathrm{C}\}} + \beta_8 x_{2i} \mathbf{1}_{\{x_{i3}=\mathrm{B}\}} + \beta_9 x_{2i} \mathbf{1}_{\{x_{i3}=\mathrm{C}\}}, \end{aligned} \tag{2}$$

via the following specification:

```
fit_2way <-
  QRRT(
    Formula = Ri ~ (x1 + x2 + as.factor(x3)) ^ 2,
    Data = Sim_Data,
    Disperse = 1,
    beta = NULL,
    n_times = 10,
    offset = NULL,
    b_distribution = c(6, 7, 4, 2, 2, 1, 1, 1, 1, 25) / 50
  )
```

Here, the `Formula` uses standard `R` syntax to specify the model with all two-way interactions; similarly,

```
fit_truemodel <-
  QRRT(
    Formula = Ri ~ (x1 + x2) ^ 2 + as.factor(x3),
    Data = Sim_Data,
```

```
    Disperse = 1,
    beta = NULL,
    n_times = 10,
    offset = NULL,
    b_distribution = c(6, 7, 4, 2, 2, 1, 1, 1, 1, 25) / 50
  )
```

would specify the true model (1). Other examples, including the use of an offset, accompany the code.

Next, `Data` specifies a data frame `Sim_Data` consisting of the three covariates $\{x_{1i}\}$, $\{x_{2i}\}$, $\{x_{3i}\}$ and the observed responses $\{r_i\}$. Because the starting value is specified as `beta = NULL`, the code selects `n_times = 10` different random starts for the $\boldsymbol{\beta}$ coefficients, using independent normal random variables with mean zero and standard deviation `Disperse = 1`. The `offset` is not used in this example, but takes its default null value (a vector of zeroes on the logarithmic scale) The `b_distribution` argument specifies the $b(r)$ distribution from

$$(b(0), b(1), \ldots, b(8), b(9)) = \frac{1}{50}(6, 7, 4, 2, 2, 1, 1, 1, 1, 25). \tag{3}$$

The code then runs the EM algorithm to convergence from each random start, finally returning the fitted model with highest likelihood:

```
                     Estimate Std.Error t-statistic Pr(>|t|)
(Intercept)             1.397     0.152       9.205   3.41e-20
x1                      1.047     0.145       7.223   5.08e-13
x2                     -0.557     0.073      -7.663   1.82e-14
as.factor(x3)B          0.644     0.183       3.528   4.18e-04
as.factor(x3)C          0.504     0.183       2.751   0.006
x1:x2                   0.203     0.062       3.279   0.001
x1:as.factor(x3)B      -0.164     0.173      -0.948   0.343
x1:as.factor(x3)C      -0.122     0.175      -0.698   0.485
x2:as.factor(x3)B       0.058     0.051       1.126   0.260
x2:as.factor(x3)C       0.039     0.052       0.757   0.449
```

The `Estimate` column of the above output shows point estimates of the true regression coefficients

$$(\beta_0, \ldots, \beta_5, \beta_6, \ldots, \beta_9) = (1.5, 1.0, -0.5, 0.4, 0.3, 0.2, 0, 0, 0, 0),$$

with excellent agreement relative to the asymptotic standard errors (`Std.Error`). That is, the fitted model correctly identifies the non-zero coefficients $(\beta_0, \ldots, \beta_5)$, with large `t-statistic` (estimate over standard error) and small p-values (`Pr(>|t|)`) and gives point estimates consistent with the true values. It also correctly identifies the zero coefficients, $(\beta_6, \ldots, \beta_9)$, with small t-statistics and large p-values.

To test the hypothesis that model (1) fits as well as model (2), we compute the log-likelihood ratio via

```
-2 * fit_true$Maximized_Log_Likelihood
    + 2 * fit_2way$Maximized_Log_Likelihood.
```

The resulting test statistic is 1.82364, with corresponding p-value of 0.7681545, computed via

```
1 - pchisq(q = 1.82364, df = 4)
```

from the χ^2 distribution with 4 degrees of freedom.

The Monte Carlo experiment of this paper repeats the above simulation, estimation and hypothesis test 1000 times. There is no evidence to reject the null hypothesis that model (1) fits as well as model (2).

References

[1] R Core Team. R: A Language and Environment for Statistical Computing; 2017. Available from: https://www.R-project.org/.
