# Supplementary material for: Understanding the drivers of sensitive behavior using Poisson regression from quantitative randomized response technique data
Source: PLoS One. 2018 Sep 28;13(9):e0204433. doi: 10.1371/journal.pone.0204433 (PMC6161884; doi:10.1371/journal.pone.0204433)
Supplement: S2 File — (PDF) [file pone.0204433.s005.pdf]

S2 FILE: SURVEY QUESTIONNAIRES

MENG CAO, F. JAY BREIDT, JENNIFER N. SOLOMON, ABU CONTEH, MICHAEL C. GAVIN

Survey questionnaires from [1], reproduced for convenient reference.

Appendix IVa: Survey Schedule
[English version]

TE WHARE WĀNANGA O TE ŪPOKO O TE IKA A MAUI
VICTORIA
UNIVERSITY OF WELLINGTON

SURVEY INSTRUMENT FOR A Ph.D. STUDY ON

Impact of war on biodiversity conservation in the Western Area Peninsula Forest Reserve, Sierra Leone

BY

ABU CONTEH

School of Geography, Environment, and Earth Sciences
Victoria University of Wellington
New Zealand

---

1. Survey administrator's code:

2. Date:

3. Name of community:

4. What is your age?

5. How long has your household been settled in this community?

6. Start time:

Part 1 – Survey on Conservation Capacity & Hunting

Section A – Perceptions of the reserve

The first set of questions will focus on your perceptions of the forest reserve adjacent to this community over three time periods. Please answer these questions with yes, no, or don't know, to the best of your knowledge. I only want to know what you think. There is no problem if you do not know an answer to a question. You only need to let me know. Sometimes, I will also ask you to tell me the reason why you answered the way you did. There are no right or wrong answers. Whatever answer you provide is your opinion and will be considered valid. You only need to answer honestly, to the best of your knowledge. If you do not understand a question, please let me know so that I can repeat it.

7a. **Prior to** the war in Freetown (1992 – 1997), did a **protected area exist** near this community?
   1. Yes
   2. No
   3. Don't know
   If yes, give the name(s)

b. **During** the war in Freetown (1997 – 2002), did a **protected area exist** near this community?
   1. Yes
   2. No
   3. Don't know
   If yes, give the name(s)

1

c. **After** the war in Freetown (2002 – 2007), did a **protected area exist** near this community?
1. Yes
2. No
3. Don't know
If yes, give the name(s)

8a. **Prior to** the war in Freetown (1992 – 1997), did you **use the resources** in the forest reserve adjacent to this community?
1. Yes
2. No
3. Don't know
If yes, list these resources

b. **During** the war in Freetown (1997 – 2002), did you **use the resources** in the forest reserve adjacent to this community?
1. Yes
2. No
3. Don't know
If yes, list these resources

c. **After** the war in Freetown (2002 – 2007), did you **use the resources** in the forest reserve adjacent to this community?
1. Yes
2. No
3. Don't know
If yes, list these resources

Now I am going to read you some statements about the conservation capacity in the reserve. Please tell me whether you strongly agree, agree, are neutral, disagree or strongly disagree. Sometimes, I will also ask you to tell me the reason why you answered the way you did. There are no right or wrong answers. I am only asking you for your opinion. If you do not understand a question, please ask me to repeat it.

9a. **Prior to** the war in Freetown (1992 – 1997), there was **no restriction posed by reserve personnel** on the **extraction** of resources in the forest reserve adjacent to this community.
1. Strongly agree
2. Agree
3. Neutral
4. Disagree
5. Strongly disagree
Why did you answer that way?

b. **During** the war in Freetown (1997 – 2002), there was **no restriction posed by reserve personnel** on the **extraction** of resources in the forest reserve adjacent to this community.

1. Strongly agree
2. Agree
3. Neutral
4. Disagree
5. Strongly disagree

Why did you answer that way?

c. **After the war in Freet**own (2002 – 2007), there was no **restriction posed by reserve personnel** on the **extraction** of resources in the forest reserve adjacent to this community.

1. Strongly agree
2. Agree
3. Neutral
4. Disagree
5. Strongly disagree

Why did you answer that way?

Section B – Perceptions of the role played by conservation personnel

The next sets of questions are about your perception of the role played by conservation personnel. Please tell me whether you strongly agree, agree, are neutral, disagree, or strongly disagree. Again, sometimes, I will also ask you to tell me the reason why you answered the way you did.

10a. **Prior to** the war in Freetown (1992 – 1997), conservation personnel were **efficient** in their duties in the forest reserve adjacent to this community.

1. Strongly agree
2. Agree
3. Neutral
4. Disagree
5. Strongly disagree

Why did you answer that way?

b. **During** the war in Freetown (1997 – 2002), conservation personnel were **efficient** in their duties in the forest reserve adjacent to this community.

1. Strongly agree
2. Agree
3. Neutral
4. Disagree
5. Strongly disagree

Why did you answer that way?

c. **After** the war in Freetown (2002 – 2007), conservation personnel were **efficient** in their duties in the forest reserve adjacent to this community.
1. Strongly agree
2. Agree
3. Neutral
4. Disagree
5. Strongly disagree
Why did you answer that way?

Section C - Personnel's dedication to duty

I will now ask you some questions about the conservation personnel's dedication to duty. Please tell me whether you strongly agree, agree, are neutral, disagree or strongly disagree. Again, sometimes I will also ask you to tell me the reason why you answered the way you did.

11a. Prior **to** the war in Freetown (1992 – 1997), conservation personnel **patrolled** the forest reserve adjacent to this community.
1. Strongly agree
2. Agree
3. Neutral
4. Disagree
5. Strongly disagree

If strongly agree or agree, was the patrol adequate for biodiversity conservation in the reserve?
1. Yes
2. No
3. Don't know
Why did you answer that way?

b. **During** the war in Freetown (1997 – 2002), conservation personnel **patrolled** the forest reserve adjacent to this community.
1. Strongly agree
2. Agree
3. Neutral
4. Disagree
5. Strongly disagree

If strongly agree or agree, was the patrol adequate for biodiversity conservation in the reserve?
1. Yes
2. No
3. Don't know
Why did you answer that way?

c. **After** the war in Freetown (2002 – 2007), conservation personnel **patrolled** the forest reserve adjacent to this community.
1. Strongly agree
2. Agree
3. Neutral
4. Disagree
5. Strongly disagree

If strongly agree or agree, was the patrol adequate for biodiversity conservation in the reserve?
1. Yes
2. No
3. Don't know

Why did you answer that way?

12a. **Prior to** the war in Freetown (1992 – 1997), you **were satisfied** with the work of the conservation personnel who were responsible for manning the forest reserve adjacent to this community.
1. Strongly agree
2. Agree
3. Neutral
4. Disagree
5. Strongly disagree

Why did you answer that way?

b. **During** the war in Freetown (1997 – 2002), you **were satisfied** with the work of the conservation personnel who were responsible for manning the forest reserve adjacent to this community.
1. Strongly agree
2. Agree
3. Neutral
4. Disagree
5. Strongly disagree

Why did you answer that way?

c. **After** the war in Freetown (2002 – 2007), you **were satisfied** with the work of the conservation personnel who were responsible for manning the forest reserve adjacent to this community.
1. Strongly agree
2. Agree
3. Neutral
4. Disagree
5. Strongly disagree

Why did you answer that way?

13a. **Prior to** the war in Freetown (1992 – 1997), conservation personnel were **quick to apprehend** people who entered the forest reserve adjacent to this community to extract resources.
1. Strongly agree
2. Agree
3. Neutral
4. Disagree
5. Strongly disagree

b. **During** the war in Freetown (1997 – 2002), conservation personnel were **quick to apprehend** people who entered the forest reserve adjacent to this community to extract resources.

1. Strongly agree
2. Agree
3. Neutral
4. Disagree
5. Strongly disagree

c. **After** the war in Freetown (2002 – 2007), conservation personnel were **quick to apprehend** people who entered the forest reserve adjacent to this community to extract resources.

1. Strongly agree
2. Agree
3. Neutral
4. Disagree
5. Strongly disagree

14a. **Prior to** the war in Freetown (1992 – 1997), conservation personnel ensured that people who were caught extracting resources in the forest reserve adjacent to this community were **punished**.

1. Strongly agree
2. Agree
3. Neutral
4. Disagree
5. Strongly disagree

If strongly agree or agree, list these punishments.

b. **During** the war in Freetown (1997 – 2002), conservation personnel ensured that people who were caught extracting resources in the forest reserve adjacent to this community were **punished**.

1. Strongly agree
2. Agree
3. Neutral
4. Disagree
5. Strongly disagree

If strongly agree or agree, list these punishments.

c. **After** the war in Freetown (2002 – 2007), conservation personnel ensured that people who were caught extracting resources in the forest reserve adjacent to this community were **punished**.

1. Strongly agree
2. Agree
3. Neutral
4. Disagree
5. Strongly disagree

If strongly agree or agree, list these punishments.

Section D - Hunting in the reserve

I am going to ask you questions about your perception of hunting in the forest adjacent to this community. Please answer the following questions with yes, no, or don't know, to the best of your knowledge.

15a. **Prior to** the war in Freetown (1992 – 1997), **people residing outside this community hunted** in the forest reserve adjacent to this community.

1. Yes
2. No
3. Don't know

If yes, where are they from?

b. **During** the war in Freetown (1997 – 2002), **people residing outside this community hunted** in the forest reserve adjacent to this community.

1. Yes
2. No
3. Don't know

If yes, where are they from?

c. **After** the war in Freetown (2002 – 2007), **people residing outside this community hunted** in the forest reserve adjacent to this community?

1. Yes
2. No
3. Don't know

If yes, where are they from?

16a. **Prior to** the war in Freetown (1992 – 1997), **residents of this community hunted in other parts of the forest reserves located outside** this community.

1. Yes
2. No
3. Don't know

If yes, where else and why?

b. **During** the war in Freetown (1997 – 2002), **residents of this community hunted in other parts of the forest reserves located outside** this community.

1. Yes
2. No
3. Don't know

If yes, where else and why?

c. **After** the war in Freetown (2002 – 2007), **residents of this community hunted in other parts of the forest reserves located outside** this community.

1. Yes
2. No
3. Don't know

If yes, where else and why?

I am going to read you statements about the animals hunted in the forest reserve adjacent to this community. Please tell me whether you strongly agree, agree, are neutral, disagree or strongly disagree.

17a. Animals that were once **common** in the forest reserve adjacent to this community **became rare, prior to** the war (1992 – 1997) in Freetown.

1. Strongly agree
2. Agree
3. Neutral
4. Disagree
5. Strongly disagree

If strongly agree or agree, list these animals.

b. Animals that were once **common** in the forest reserve adjacent to this community **became rare, during** the war (1997 – 2002) in Freetown.

1. Strongly agree
2. Agree
3. Neutral
4. Disagree
5. Strongly disagree

If strongly agree or agree, list these animals.

c. Animals that were once **common** in the forest reserve adjacent to this community **became rare, after** the war (2002 – 2007) in Freetown.

1. Strongly agree
2. Agree
3. Neutral
4. Disagree
5. Strongly disagree

If strongly agree or agree, list these animals.

18. Overall, how has hunting changed from before, to during, and then after, the war?

Part 2 - RANDOMIZED RESPONSE TECHNIQUE

Now, I will like to ask you personal questions about hunting and other activities in the reserve. I will use a procedure that will protect your answer and keep it secret. There will be no way your answers could be attributable to you individually or the questions that you have answered. Therefore, there should be no fear of potential embarrassment and stigma related to these questions on hunting. Hence, there should be no need to conceal or falsify the facts.

Section E – Prevalence of Hunting and other activities in the reserve

Pair-wise Alternate Method

First, I need you to flip this coin like this *[Survey administrator will demonstrate coin flipping*] and then look at what it lands on. There are two choices, it may land showing you a head, or it may land showing you a tree. Don't tell me what it lands on. You only need to remember what it lands on and keep it as your secret.

Next, I am going to show you two pictures. One is of a coin with a head on it, similar to the one you may see on the coin. On another, is a picture depicting an activity (firewood collection, charcoal burning, medicinal plants collection, hunting, etc) you may have done, or one of the methods that may have been used to hunt (shot guns, local traps/snares, hunting dogs, trenches), in the forest reserve adjacent to this community. For each question, I will place the picture of the coin and that showing the activity in question in a bag. You will choose one of the pictures. It is random. I don't know which one you will choose.

Look at the picture. Don't tell me which one you are looking at. If you are looking at the head, tell me "yes" if you saw head when you flipped the coin. If you saw the tree, then, tell me "no". If you are looking at the picture (showing the activity in question), tell me yes, if you or any member of your household ever went to the reserve to do the activity, or if you used the hunting method, as depicted in the picture, during the period the question refers to. If no member of your household ever went to the reserve to do the activity, or used the hunting method, as depicted in the picture, tell me no. Please be honest with me, as I have no way of knowing which picture you are looking at.

[A trial run using role reversal will be performed]

Now, without telling me what you get, please flip the coin and answer yes or no to the picture you pick.

A picture depicting the head of a coin and another depicting **firewood collection** in the forest reserve adjacent to this community during the following periods:
19a. **Prior to** the war in Freetown (1992 – 1997)……………………………………………………
b. **During** the war in Freetown (1997 – 2002) ……………………………………………………
c. **After** the war in Freetown (2002 – 2007) ……………………………………………………

A picture depicting the head of a coin and another depicting **charcoal production** in the forest reserve adjacent to this community during the following periods:
20a. **Prior to** the war in Freetown (1992 – 1997)……………………………………………………
b. **During** the war in Freetown (1997 – 2002) ……………………………………………………
c. **After** the war in Freetown (2002 – 2007) ……………………………………………………

A picture depicting the head of a coin and another depicting **hunting** in the forest reserve adjacent to this community during the following periods:
21a. **Prior to** the war in Freetown (1992 – 1997)……………………………………………………
b. **During** the war in Freetown (1997 – 2002) ……………………………………………………
c. **After** the war in Freetown (2002 – 2007) ……………………………………………………

A picture depicting the head of a coin and another depicting the **use of shot-guns to hunt** in the forest reserve adjacent to this community during the following periods:
22a. **Prior to** the war in Freetown (1992 – 1997)……………………………………………………
b. **During** the war in Freetown (1997 – 2002) ……………………………………………………
c. **After** the war in Freetown (2002 – 2007) ……………………………………………………

A picture depicting the head of a coin and another depicting the **use of snares/local traps** to hunt/trap in the forest reserve adjacent to this community during the following periods:

23a. **Prior to** the war in Freetown (1992 – 1997)……………………………………………………………
b. **During** the war in Freetown (1997 – 2002) ……………………………………………………………
c. **After** the war in Freetown (2002 – 2007) ………………………………………………………………

A picture depicting the head of a coin and another depicting the **live traps** in the forest reserve adjacent to this community during the following periods:

24a. **Prior to** the war in Freetown (1992 – 1997)……………………………………………………………
b. **During** the war in Freetown (1997 – 2002) ……………………………………………………………
c. **After** the war in Freetown (2002 – 2007) ………………………………………………………………

A picture depicting the head of a coin and another depicting the **use of net and hunting dogs to hunt** in the forest reserve adjacent to this community during the following periods:

25a. **Prior to** the war in Freetown (1992 – 1997)……………………………………………………………
b. **During** the war in Freetown (1997 – 2002) ……………………………………………………………
c. **After** the war in Freetown (2002 – 2007) ………………………………………………………………

A picture depicting the head of a coin and another depicting the **use of trenches to hunt** in the forest reserve adjacent to this community during the following periods:

26a. **Prior to** the war in Freetown (1992 – 1997)……………………………………………………………
b. **During** the war in Freetown (1997 – 2002) ……………………………………………………………
c. **After** the war in Freetown (2002 – 2007) ………………………………………………………………

Section F – Intensity of Hunting in the reserve

The Quantitative Forced Alternative Randomizing Device Method

Now, I will ask some questions about the number of times your households went hunting by trapping and by use of shot guns in the forest reserve adjacent to this community and the number of animals obtained per trip in each case. In this container, there are an equal number of orange and green balls. The green balls all have numbers printed upon them. As you can see, the numbers range from zero to eight. By inverting the container, one ball drops into the neck.

I will be asking you a few questions. For each question, reflect on how many times per month this year (during the nine month period under question - 1st January, 2008 to 1st October, 2008), you or your household ever went hunting in the forest reserve adjacent to this community, or the number of animals obtained per trip of hunting. Don't tell me the answer now. You only need to keep it in mind for now. You may or may not say it, depending on which ball enters the bottle neck.

Now, shake the bottle well in order to mix up the balls. I will turn around so that I can't see which colour ball enters the bottle neck. Then, invert the bottle so that a ball drops into the neck. If a green ball is housed in the bottle neck, tell me the number printed on the ball. If the housed ball is orange, tell me the correct answer to the question which you kept in mind. If the answer is greater than 8, then answer 8. Please supply the answer as a number e.g. 0, 1, 2, 3, etc. Avoid using words such as none or once so that I can't tell whether you are reading the number on the ball or answering the question.

[A trial run using role reversal will be performed]

Now, we are ready to begin.

27. **In 2008** (New Year's Day to Pray day [1st Oct]), **how many times per month** did you or any member of your household **hunt by trapping** in the forest reserve adjacent to this community? ....

28. **In 2008** (New Year's Day to Pray day [1st Oct]), **how many times per year** did you or any member of your household **hunt with the use of shot-guns** in the forest reserve adjacent to this community? ......

29. **In 2008** (New Year's Day to Pray day [1st Oct]), about **how many animals** did you or any member of your household get **per trip of hunting**, when hunting **by trapping**? ....

30. **In 2008** (New Year's Day to Pray day [1st Oct]), about **how many animals** did you or any member of your household get **per trip of hunting**, when hunting with **shot-guns**? ....

Section G - Socio-Demographic data

Finally, I will now ask you questions about yourself and your household.

31. Respondent's address:

32. Gender
    1. Male
    2. Female

33. What is your religion?
    1. Islam
    2. Christianity
    3. Traditional
    4. None
    5. Others (please specify).......................................

34. What is your marital status?
    1. Single
    2. Married
    3. Widowed
    4. Divorced
    5. Partnership

35. What is the highest level of education you have attained?
    1. None
    2. Primary
    3. Junior Secondary / GCE Ordinary Level
    4. Senior Secondary / GCE Advanced Level
    5. Technical/Vocational
    6 Quranic

36. What is your tribe?

37. How many people are in your household?

38. What is your occupation?

End time:
Thank you for your time and patience in this survey.

Appendix IVb: Survey Schedule
[Krio version]

TE WHARE WĀNANGA O TE ŪPOKO O TE IKA A MĀUI
VICTORIA
UNIVERSITY OF WELLINGTON

SORVAE PAEPA FOR ONE Ph.D. STORDI

D wah ein amburgin pan de bush en de beef den na de Peninsula Forest na westen area ya, Salone

Bi

ABU CONTEH

Skol fo Gografi, Enviroment, en Earth Sciences
Viktoria Univasity na Welintin
New Zealand

---

1. Di kod fo di sorveyo:

2. Tidae date:

3. Dis komuniti en naem:

4. Omos iya u ol?

5. Aw long u don tap na dis komuniti?

6. De tem wae we begin de sorvae:

Pat 1 – Sorvae bot aw u kin manej di konsaveshon en ontin

Sekshon A – Aw pipul den fil bot di rizav

Di fos kweshon den go luk aw u de fil bɔt de forest rizav wae de nia dis komuniti fo di pas tri iya. Duya, na fo mek ar kno if u tru tru ansa na yes, nor, or ar nor no fo di kweshon den wae ar go ask. Wae u de ansa, ar wan sabi normor wetin u tink. Problem nor de if u nor kno di ansa fo ayni kweshon. Jus mek ar kno. Sontem dae, ar go ask u fo tel me waetin mek u ansa lek dat. Wi nor get ayni korekt or rong ansa fo ayni kweshon. Ayni ansa wae u gi mi dae reflect u yon opinion en ar go taek am lek tru. U jus get fo tok di tru. If u nor ɔndastand ayni kweshon, duya tel mi fo mek ar ripit am.

7a. **Bifo** di wa na Freton (1992 – 1997), una bin get anisai nia dis komuniti wae **pipul de protekt**?
1. Yes
2. Nor
3. Ar nor kno
If u ansa na yes, gi mi di naem (dem)

b. **Durin** di wa na Freton (1997 – 2002), una bin get anisai nia dis komuniti wae **pipul de protekt**?
1. Yes
2. Nor
3. Ar nor kno
If u ansa na yes, gi mi di naem (dem)

c. **Afta** di wa na Freton (2002 – 2007), una bin get anisai nia dis komuniti wae **pipul de protekt**?

1. Yes
2. Nor
3. Ar nor kno

If u ansa na yes, gi mi di naem (dem)

8a. **Bifo** di wa na Freton (1992 – 1997), u bin **de uz aynitin** wae dae **na di forest** nia dis komuniti?

1. Yes
2. Nor
3. Ar nor kno

If u ansa na yes, gi di naem fo den tin den dae

b. **Durin** di wa na Freton (1997 – 2002), u bin **de uz aynitin** wae dae **na di forest** nia dis komuniti?

1. Yes
2. Nor
3. Ar nor kno

If u ansa na yes, gi di naem of den tin den dae

c. **Afta** di wa na Freton (2002 – 2007), u bin **de uz aynitin** wae dae **na di forest** nia dis komuniti?

1. Yes
2. Nor
3. Ar nor kno

If u ansa na yes, gi di naem of den tin den dae

Naw, ar go rid to u sontin dem wae de tok bɔt di konsaveshon kepasiti na di rizav. Duya tel mi if u gri tranga tranga wan, u gri or u nor de ya or yanda, u nor gri, u nor gri at ɔl at ɔl. Sontem dae, ar go ask u di rizin wae mek u gi da ansa de. Wi nor get ayni ansa wae korekt or rong. Na u idiya normo ar wan fo get. If u nor ɔndastand ayni kweshon, duya ask mi fo mek ar ripit am.

9a. **Bifo** di wa na Freton (1992 – 1997), **di wokman den for dis rizav** nor bin **de stop una fo taek tin den** na dis forest wae de niya dis komuniti.

1. Ar gri tranga tranga wan
2. Ar gri
3. Ar nor de ya or yanda
4. Ar nor gri
5. Ar nor gri at ɔl at ɔl

Waetin mek u sae so?

b. **Durin** di wa na Freton (1997 – 2002), **di wokman den for dis rizav** nor bin **de stop una fo taek tin den** na dis forest wae dae niya dis komuniti.

1. Ar gri tranga tranga wan
2. Ar gri
3. Ar nor de ya or yanda
4. Ar nor gri
5. Ar nor gri at ɔl at ɔl

Waetin mek u sae so?

c. **Afta** di wa na Freton (2002 – 2007), **di wokman den for dis rizav** nor bin **de stop una fo taek tin den** na dis forest wae de niya dis komuniti.

1. Ar gri tranga tranga wan
2. Ar gri
3. Ar nor de ya or yanda
4. Ar nor gri
5. Ar nor gri at ɔl at ɔl

Waetin mek u sae so?

Sekshon B – Ow u fil bot de wok wae de konsaveshon wokman den dae do na dis rizav

De nex kweshon den wae ar dae can ask u na bɔt waetin u fil bɔt de wok wae de konsaveshon wokman den dae do na dis rizav. Duya tel mi if u gri tranga tranga wan, u gri or u nor de ya or yanda, u nor gri, u nor gri at ɔl at ɔl. Aegain, sontem dae, ar go ask u fo tel mi di rizin wae mek u gi da ansa dae.

10a. **Bifo** di wa na Freton (1992 – 1997), de konsaveshon wokman den bin dae wok **bête bête** wan na dis rizav neiba dis komuniti.

1. Ar gri tranga tranga wan
2. Ar gri
3. Ar nor de ya or yanda
4. Ar nor gri
5. Ar nor gri at ɔl at ɔl

Waetin mek u sae so?

b. **Durin** di wa na Freton (1997 – 2002), de konsaveshon wokman den bin dae wok **bête bête** wan na dis rizav neiba dis komuniti.

1. Ar gri tranga tranga wan
2. Ar gri
3. Ar nor de ya or yanda
4. Ar nor gri
5. Ar nor gri at ɔl at ɔl

Waetin mek u sae so?

c. **Afta** di wa na Freton (2002 – 2007), de konsaveshon wokman den bin dae wok **bête bête** wan na dis rizav neiba dis komuniti

1. Ar gri tranga tranga wan
2. Ar gri
3. Ar nor de ya or yanda
4. Ar nor gri
5. Ar nor gri at ɔl at ɔl

Waetin mek u sae so?

Sekshon C – Ow de konsaveshon wokman den taek de wok

Naw, ar go ask u som kweshon den bot ow de konsaveshon wokman den taek dem wok. Duya tel mi if u gri tranga tranga wan, u gri or u nor de ya or yanda, u nor gri, u nor gri at ɔl at ɔl. Aegain, sontem dae, ar go ask u fo tel mi di rizin wae mek u gi da ansa dae.

11a. **Bifo** di wa na Freton (1992 – 1997), de konsaveshon wokman den bin dae **patrɔl** na de rizav neiba dis komuniti.

1. Ar gri tranga tranga wan
2. Ar gri
3. Ar nor de ya or yanda
4. Ar nor gri
5. Ar nor gri at ɔl at ɔl

If u gri tranga tranga wan, or u gri, tel mi if de patrɔl bin gud fo de bush en fo den bif den insae dae?

1. Yes
2. Nor
3. Ar nor kno

Waetin mek u sae so?

b. **Durin** di wa na Freton (1997 – 2002), de konsaveshon wokman den bin dae **patrɔl** na de rizav neiba dis komuniti.

1. Ar gri tranga tranga wan
2. Ar gri
3. Ar nor de ya or yanda
4. Ar nor gri
5. Ar nor gri at ɔl at ɔl

If u gri tranga tranga wan, or u gri, tel mi if de patrɔl bin gud fo de bush en fo den bif den insae dae?

1. Yes
2. Nor
3. Ar nor kno

Waetin mek u sae so?

c. **Afta** di wa na Freton (2002 – 2007), d konsaveshon wokman den bin dae **patrɔl** na dis rizav neiba dis komuniti.

1. Ar gri tranga tranga wan
2. Ar gri
3. Ar nor de ya or yanda
4. Ar nor gri
5. Ar nor gri at ɔl at ɔl

If u gri tranga tranga wan, or u gri, tel me if de patrɔl bin gud fo de bush en fo den bif den insae dae?

1. Yes
2. Nor
3. Ar nor kno

Waetin mek u sae so?

12a. **Bifo** di wa na Freton (1992 – 1997), u **bin satisfy** wit de wok wae de konsaveshon wokman den bin dae do na de rizav neiba dis komuniti.

1. Ar gri tranga tranga wan
2. Ar gri
3. Ar nor de ya or yanda
4. Ar nor gri
5. Ar nor gri at ɔl at ɔl

Waetin mek u sae so?

b. **Durin** di wa na Freton (1997 – 2002), u **bin satisfy** wit de wok wae de konsaveshon wokman den bin dae do na de rizav neiba dis komuniti.

1. Ar gri tranga tranga wan
2. Ar gri
3. Ar nor de ya or yanda
4. Ar nor gri
5. Ar nor gri at ɔl at ɔl

Waetin mek u sae so?

c. **Afta** di wa na Freton (2002 – 2007), u **bin satisfy** wit de wok wae de konsaveshon wokman den bin dae do na de rizav neiba dis komuniti.

1. Ar gri tranga tranga wan
2. Ar gri
3. Ar nor de ya or yanda
4. Ar nor gri
5. Ar nor gri at ɔl at ɔl

Waetin mek u sae so?

13a. **Bifo** di wa na Freton (1992 – 1997), de konsaveshon wokman den bin **quik fo ɔl** pipul dem wae bin dae go na de rizav neiba dis komuniti fo go pɔl tin dem.

1. Ar gri tranga tranga wan
2. Ar gri
3. Ar nor de ya or yanda
4. Ar nor gri
5. Ar nor gri at ɔl at ɔl

b. **Durin** di wa na Freton (1997 – 2002), de konsaveshon wokman den bin **quik fo ɔl** pipul dem wae bin dae go na de rizav neiba dis komuniti fo go pɔl tin dem.

1. Ar gri tranga tranga wan
2. Ar gri
3. Ar nor de ya or yanda
4. Ar nor gri
5. Ar nor gri at ɔl at ɔl

c. **Afta** di wa na Freton (2002 – 2007), de konsaveshon wokman den bin **quik fo ɔl** pipul dem wae bin dae go na de rizav neiba dis komuniti fo go pɔl tin dem.

1. Ar gri tranga tranga wan
2. Ar gri
3. Ar nor de ya or yanda
4. Ar nor gri
5. Ar nor gri at ɔl at ɔl

14a. **Bifo** di wa na Freton (1992 – 1997), de konsaveshon wokman den bin dae maek shor sae **den pɔnish** pipul wae den kaytch dae go na de rizav neiba dis komuniti fo go pɔl tin dem.

1. Ar gri tranga tranga wan
2. Ar gri
3. Ar nor de ya or yanda
4. Ar nor gri
5. Ar nor gri at ɔl at ɔl

If u gri tranga tranga wan, or u gri, tel me de punishment dem.

b. **Durin** di wa na Freton (1997 – 2002), de konsaveshon wokman den bin dae maek shor sae **den pɔnish** pipul wae den kaytch dae go na de rizav neiba dis komuniti fo go pɔl tin dem.

1. Ar gri tranga tranga wan
2. Ar gri
3. Ar nor de ya or yanda
4. Ar nor gri
5. Ar nor gri at ɔl at ɔl

If u gri tranga tranga wan, or u gri, tel me de punishment dem.

c. **Afta** di wa na Freton (2002 – 2007), de konsaveshon wokman den bin dae maek shor sae **den pɔnish** pipul wae den kaytch dae go na de rizav neiba dis komuniti fo go pɔl tin dem.

1. Ar gri tranga tranga wan
2. Ar gri
3. Ar nor de ya or yanda
4. Ar nor gri
5. Ar nor gri at ɔl at ɔl

If u gri tranga tranga wan, or u gri, tel me de punishment dem.

Sekshon D - Ontin na di rizav

Ar dae can ask u kweshon bɔt waetin u fil arbawt ontin na dis rizav neiba dis komuniti. Duya, na fo maek ar kno if u tru tru ansa na yes, nor, or a nor no.

15a. **Bifo** di wa na Freton (1992 – 1997), **pipul wae nor tap na dis komuniti, bin dae ontin** na de rizav neiba dis komuniti.

1. Yes
2. Nor
3. Ar nor kno

If u ansa sae yes, usai dem bin dae comot?

b. **Durin** di wa na Freton (1997 – 2002), **pipul wae nor tap na dis komuniti, bin dae ontin** na de rizav neiba dis komuniti.

1. Yes
2. Nor
3. Ar nor kno

If u ansa sae yes, usai dem bin dae comot?

c. **Afta** di wa na Freton (2002 – 2007), **pipul wae nor tap na dis komuniti, bin dae ontin** na de rizav neiba dis komuniti

1. Yes
2. Nor
3. Ar nor kno

If u ansa sae yes, usai dem bin dae comot?

16a. **Bifo** di wa na Freton (1992 – 1997), **pipul wae bin lib na dis komuniti bin dae go ontin far way na oda part dem**.

1. Yes
2. Nor
3. Ar nor kno

If u ansa sae yes, usai aegain en waetin du?

b. **Durin** di wa na Freton (1997 – 2002), **pipul wae bin lib na dis komuniti bin dae go ontin far way na oda part dem**.

1. Yes
2. Nor
3. Ar nor kno

If u ansa sae yes, usai aegain en waetin du?

c. **Afta** di wa na Freton (2002 – 2007), **pipul wae bin lib na dis komuniti bin dae go ontin far way na oda part dem**.

1. Yes
2. Nor
3. Ar nor kno

If u ansa sae yes, usai aegain en waetin du?

Ar go rid som staitment fo u wae na arbawt de animal dem wae pipul dae ontin na de rizav neiba dis komuniti. Duya tel mi if u gri tranga tranga wan, u gri, u nor de ya or yanda, u nor gri, u nor gri at ɔl at ɔl wit de staitment dem.

17a. Den bif wae bin **plenty** tradae na de rizav neiba dis komuniti, kan art fo see jus **bifo** di wa (1992 – 1997) na Freton.

1. Ar gri tranga tranga wan
2. Ar gri
3. Ar nor de ya or yanda
4. Ar nor gri
5. Ar nor gri at ɔl at ɔl

If u gri tranga tranga wan, or u gri, naem de animal dem.

b. Den bif wae bin **plenty** tradae na de rizav neiba dis komuniti, kan art fo see **wa tem** (1997 – 2002) na Freton.

1. Ar gri tranga tranga wan
2. Ar gri
3. Ar nor de ya or yanda
4. Ar nor gri
5. Ar nor gri at ɔl at ɔl

If u gri tranga tranga wan, or u gri, naem de animal dem.

c. Den bif wae bin **plenty** tradae na de rizav neiba dis komuniti, kan art fo see**, afta** di wa (2002 – 2007) na Freton.

1. Ar gri tranga tranga wan
2. Ar gri
3. Ar nor de ya or yanda
4. Ar nor gri
5. Ar nor gri at ɔl at ɔl

If u gri tranga tranga wan, or u gri, naem de animal dem.

18. Tok go, tok kam, ow ontin bin dae bifo di wa, wa tem, en afta de wa?

Pat 2 - <u>RANDOMIZED RESPONS TEKNIK</u>

Naw, ar go lek fo ask u pasonal kweshon arbawt ontin en ɔda wok dem na de rizav. Ar go uze wan metod wae go coba u ansa. Non wae nor dae wae anibodi go kno sae na u ansa dat or sae na da kweshon dae eehn ansa dat. So, u nor get fo fraid sae ani molestation or wahala go kam bambai bekos u ansa dem kweshon ya bɔt ontin. Bekos of dat, nid no dae fo maek u hyd or coba de tru.

Sekshon E – Ow Ontin en Oda wok dem dae na de rizav

Pear-wyze Altanate Metod

Fos, ar warnt mek u flip dis corpoh lek so *[De porson wae dae lid de sorvae go show aw fo flip de corpoh*] don luk see waetin show wen de corpoh fɔdɔm. Na tu waye dae, e go fɔdɔm en de aide de sho ɔp, or e go fɔdɔm en sho de tik na de bak. Nor tel mi waetin e sho now. U jus get fo memba ram normoh fo bambai u go uz am lata na dis tok.

Don, ar go sho u tu piktɔr. Wan get de aide wae feba de wan wae you go see pan de coin. De oda wan, go sho wan pan dem ɔda wok dem (fo kut fayawud, bɔrn koal, pɔl meresin, Ontin, en ɔda wan dem) wae sontem dae u bin don do, or wan pan dem wae dem fo ontin (gon, chɔk nek trap, dɔg fo ontin, coba ol), na de rizav neiba dis komuniti. Fo ani wan pan de kweshon dem, ar go pɔt de piktɔre wae dae sho de corpoh en wan wae de sho de wok wae de kweshon dae tok bɔt insie dis bag. U go pik wan pan dem. U no go luk wae u dae pik, jus pik nor mo. Mi sef no go no os wan u go pik.

Luk de piktɔre wae u pik. Nɔ tel mi os wan u pik. If u dae luk de aide, sae "yes" if u bin see aide wen u flip de corpoh. If u bin see de tik, sae "nor". If u dae luk de piktɔre (wae dae sho de wok wae de kweshon dae tok bɔt), sae "yes", if u or ani wan pan de pipul dem na u ose bin don wan dae go na de rizav fo go do da wok dae, or if u bin uze de waye wae de piktɔre dae sho fo ontin, da tem dae. If nɔnbodi na u ose nɔ wan daye go na de rizav fo do da wok dae, or uze de waye wae de piktɔre dae sho fo ontin, sae "nor". Du ya tok de tru, bekoz nɔn waye nɔr dae wae ar go kno os piktɔre u dae luk.

[We go test fo se if we ɔndastand, dɔn we go change posishon en test agayn]

Naw, memba sae u nɔr fɔ tel mi wetin u get o, so du ya flip de corpoh en ansa "yes" or "no" fo de piktɔre wae u go pik.

Wan piktɔre wae dae sho de aide pan de corpoh en wan oda wan wae dae sho **kut fayawud** na de rizav neiba dis komuniti fo dem tem den ya:

19a. **Bifo** di wa na Freton (1992 – 1997)..
b. **Durin** di wa na Freton (1997 – 2002) ....
c. **Afta** di wa na Freton (2002 – 2007) ....

Wan piktɔre wae dae sho de aide pan de corpoh en wan oda wan wae dae sho **bɔrn koal** na de rizav neiba dis komuniti fo dem tem den ya:

20a. **Bifo** di wa na Freton (1992 – 1997)..
b. **Durin** di wa na Freton (1997 – 2002) ....
c. **Afta** di wa na Freton (2002 – 2007) ....

Wan piktɔre wae dae sho de aide pan de corpoh en wan oda wan wae dae sho **Ontin** na de rizav neiba dis komuniti fo dem tem den ya:

21a. **Bifo** di wa na Freton (1992 – 1997)..
b. **Durin** di wa na Freton (1997 – 2002) ....
c. **Afta** di wa na Freton (2002 – 2007) ....

Wan piktɔre wae dae sho de aide pan de corpoh en wan oda wan wae dae sho sae u kin uze **gon** fo ontin na de rizav neiba dis komuniti fo dem tem den ya:

22a. **Bifo** di wa na Freton (1992 – 1997)..
b. **Durin** di wa na Freton (1997 – 2002) ....
c. **Afta** di wa na Freton (2002 – 2007) ....

Wan piktɔre wae dae sho de aide pan de corpoh en wan oda wan wae dae sho sae u kin uze **chɔk nek trap** fo ontin na de rizav neiba dis komuniti fo dem tem den ya:

23a. **Bifo** di wa na Freton (1992 – 1997)..

b. **Durin** di wa na Freton (1997 – 2002) ....

c. **Afta** di wa na Freton (2002 – 2007) ....

Wan piktɔre wae dae sho de aide pan de corpoh en wan oda wan wae dae sho sae u kin uze **bɔx trap** fo ontin na de rizav neiba dis komuniti fo dem tem den ya:

24a. **Bifo** di wa na Freton (1992 – 1997)..

b. **Durin** di wa na Freton (1997 – 2002) ....

c. **Afta** di wa na Freton (2002 – 2007) ....

Wan piktɔre wae dae sho de aide pan de corpoh en wan oda wan wae dae sho sae u kin uze **net en dɔg** fo ontin na de rizav neiba dis komuniti fo dem tem den ya:

25a. **Bifo** di wa na Freton (1992 – 1997)..

b. **Durin** di wa na Freton (1997 – 2002) ....

c. **Afta** di wa na Freton (2002 – 2007) ....

Wan piktɔre wae dae sho de aide pan de corpoh en wan oda wan wae dae sho sae u kin uze **coba ol** fo ontin na de rizav neiba dis komuniti fo dem tem den ya:

26a. **Bifo** di wa na Freton (1992 – 1997)..

b. **Durin** di wa na Freton (1997 – 2002) ....

c. **Afta** di wa na Freton (2002 – 2007) ....

Sekshon F – Omos Ontin dae keri on na de rizav

De Quantitaeteev Fɔrced Altanateev Randomizin Devise Metod

Naw, ar go ask u som kweshon arbawt omos tem u or anibodi na u ose don go fo ontin (wae u uze trap o, or wae u uze gon) na de rizav neiba dis komuniti en omos beef u bin get ayni tem u go. Insie dis bɔtul, we get saym nɔmba of ɔrayng en grin bɔl dem. De grin bɔl dem ɔl get nɔmba mahk pan dem. As u se, de nɔmba dem na from zero to eit. If u tɔn de bɔtul oba, wan bɔl go rɔl go insie de bɔtul nek.

Ar go ask u som kweshon. Fo ayni wan pan de kweshon dem, ar want mek u tink bɔt lek omos tem insie wan mont insie dis yia (fo dis nayn mont ya wae we dae tok bɔt - 1st January, 2008 to 1st October, 2008), wae u or aynibodi na u ose don eva go ontin na de fɔrest neiba dis komuniti, or omos beef u bin get ayni tem u go. Memba sae u nɔr fɔ tel mi de ansa naw. U jus get fo memba ram normoh fo bambai u go uz am lata na dis tok. Sontem sef, u nɔr go uze am dependin on de bɔl wae fɔdɔm na de bɔtul nek.

Naw, shayk de bɔtul wel so dat de bɔl dem go mix up bêtê wan. Ar go tɔn me fais rɔund so dat ar nɔr go see ɔs corla bɔl go enta de bɔtul neck. Dɔn, tɔn de bɔtul oba, fo mek wan bɔl go rɔl go insie de bɔtul nek. If na grin bɔl enta de bɔtul nek, tel me de nɔmba wae mahk pan de bɔl. If na ɔrayng bɔl enta, tel me de korekt ansa fo de kweshon wae u bin kip na myind. If de ansa beig pas 8, wel stil ansa 8. Du ya uze nɔmba fo gie mi de ansa e.g. 0, 1, 2, 3, etc. Nɔr uze wɔds lek nɔne or wan tem fo mek ar nɔr go knɔ wen u dae rid from de bɔl or u dae gie me de ansa u bin kip na myind.

[We go test fo se if we ɔndastand, dɔn we go change posishon en test agayn]

Naw, wi don redi fo begin.

27. **Insie 2008** (Niu Yia Day to Pray day [1st Oct]), lek **omos tem insie wan mont** wae u or aynibodi na u ose bin go **ontin wit trap** na de fɔrest neiba dis komuniti? ....

28. **Insie 2008** (Niu Yia Day to Pray day [1st Oct]), lek **omos tem insie wan yia** wae u or aynibodi na u ose bin go **ontin wit gon** na de fɔrest neiba dis komuniti? ......

29. **Insie 2008** (Niu Yia Day to Pray day [1st Oct]), lek **omos animal** wae u or aynibodi na u ose bin dae get **ayni tem** den **set trap** for ontin? ....

30. **Insie 2008** (Niu Yia Day to Pray day [1st Oct]), lek **omos animal** wae u or aynibodi na u ose bin dae get **ayni tem** den go ontin wit **gon**? ....

Sekshon G - Infɔmeshon bɔt layf en di sɔrɔndin

Naw wae we dae end, Ar go ask u kweshon arbawt usef en oda pipul dem na u ose.

31. Waetin na dis adres:

32. Waetin u be (ar meen man or uman)
    1. Man
    2. Uman

33. Waetin na u religion?
    1. Muslim
    2. Kristen
    3. Suciety belif
    4. Ar nɔr dae ayni sie
    5. Oda wan dem (Du ya tel me ɔs wan).......................................

34. U maraid?
    1. Palampo
    2. Maraid
    3. Mi man don die / Mi wef don die
    4. Wi maraid bin don scata
    5. Tap to mi

35. Omos edukashion u get?
    1. Ar nor larn at ɔl
    2. Primary
    3. JSS / GCE O-Level
    4. SS / GCE A-Level
    5. Teknikal/Vokashional Institut
    6. Marabu

36. Waetin na u tribe?

37. Omos pipul lib na u os?

38. Os wok u dae do?

Tem wae we done this sorvae:
Tenki fo di tem en peshens wae u gi na dis sorvae.

References

[1] Conteh A. Impact of War on Biodiversity Conservation in the Western Area Peninsula Forest Reserve, Sierra Leone: A Thesis Submitted to the Victoria University of Wellington in Fulfilment of the Requirements for the Degree of Doctor of Philosophy in Environmental Studies. Victoria University of Wellington; 2010.
